# Silver trimagnesium phosphate bis­(hydrogenphosphate), AgMg_3_(PO_4_)(HPO_4_)_2_, with an alluaudite-like structure

**DOI:** 10.1107/S1600536810053304

**Published:** 2010-12-24

**Authors:** Abderrazzak Assani, Mohamed Saadi, Mohammed Zriouil, Lahcen El Ammari

**Affiliations:** a Laboratoire de Chimie du Solide Appliquée, Faculté des Sciences, Université Mohammed V-Agdal, Avenue Ibn Battouta, BP 1014, Rabat, Morocco

## Abstract

The title compound, AgMg_3_(PO_4_)(HPO_4_)_2_, which has been synthesized by the hydro­thermal method, has an alluaudite-like structure which is formed by edge-sharing MgO_6_ octa­hedra (one of which has symmetry 2), resulting in chains linked together by phosphate groups and hydrogen bonds. The three-dimensional framework leads to two different channels along the *c* axis, one of which is occupied by Ag^+^ ions with a square-planar coordination. The Ag^+^ ions are disordered over two sites in a 0.89 (3):0.11 (3) ratio. The OH groups, which point into the other type of channel, are involved in strong O—H⋯O hydrogen bonds. The title compound is isotypic with the compounds *AM*_3_H_2_(*X*O_4_)(H*X*O_4_)_2_ (*A* = Na or Ag, *M* = Mn, Co or Ni, and *X* = P or As) of the alluaudite structure type.

## Related literature

For applications of related compounds, see: Kacimi *et al.* (2005 ▶); Korzenski *et al.* (1998 ▶); Trad *et al.* (2010 ▶). For compounds with the same structure type, see: Moore (1971 ▶); Hatert (2008 ▶); Hatert *et al.* (2000 ▶); Assani *et al.* (2010 ▶); Guesmi & Driss (2002 ▶); Ben Smail & Jouini (2002 ▶); Stock & Bein (2003 ▶); Leroux *et al.* (1995 ▶).

## Experimental

### 

#### Crystal data

AgMg_3_(PO_4_)(HPO_4_)_2_
*M* *_r_* = 467.73
Monoclinic, 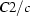

*a* = 11.9126 (5) Å
*b* = 12.1197 (6) Å
*c* = 6.4780 (3) Å
β = 113.812 (2)°
*V* = 855.66 (7) Å^3^
*Z* = 4
Mo *K*α radiation
μ = 3.21 mm^−1^
*T* = 296 K
0.31 × 0.16 × 0.12 mm

#### Data collection

Bruker X8 APEX diffractometer
Absorption correction: multi-scan (*SADABS*; Bruker, 2005 ▶) *T*_min_ = 0.545, *T*_max_ = 0.680
10680 measured reflections
2330 independent reflections
1998 reflections with *I* > 2σ(*I*)
*R*_int_ = 0.034

#### Refinement

*R*[*F* ^2^ > 2σ(*F* ^2^)] = 0.026
*wR*(*F* ^2^) = 0.075
*S* = 1.08
2330 reflections
91 parameters
H-atom parameters constrained
Δρ_max_ = 0.63 e Å^−3^
Δρ_min_ = −1.28 e Å^−3^

### 

Data collection: *APEX2* (Bruker, 2005 ▶); cell refinement: *SAINT* (Bruker, 2005 ▶); data reduction: *SAINT*; program(s) used to solve structure: *SHELXS97* (Sheldrick, 2008 ▶); program(s) used to refine structure: *SHELXL97* (Sheldrick, 2008 ▶); molecular graphics: *ORTEP-3 for Windows* (Farrugia, 1997 ▶) and *DIAMOND* (Brandenburg, 2006 ▶); software used to prepare material for publication: *WinGX* (Farrugia, 1999 ▶).

## Supplementary Material

Crystal structure: contains datablocks I, global. DOI: 10.1107/S1600536810053304/fj2371sup1.cif

Structure factors: contains datablocks I. DOI: 10.1107/S1600536810053304/fj2371Isup2.hkl

Additional supplementary materials:  crystallographic information; 3D view; checkCIF report

## Figures and Tables

**Table 1:** Hydrogen-bond geometry (Å, °)

*D*—H⋯*A*	*D*—H	H⋯*A*	*D*⋯*A*	*D*—H⋯*A*
O6—H6⋯O1^i^	0.86	1.68	2.5266 (17)	168

Symmetry code: (i) 
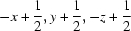
.

**Fig. 1.**
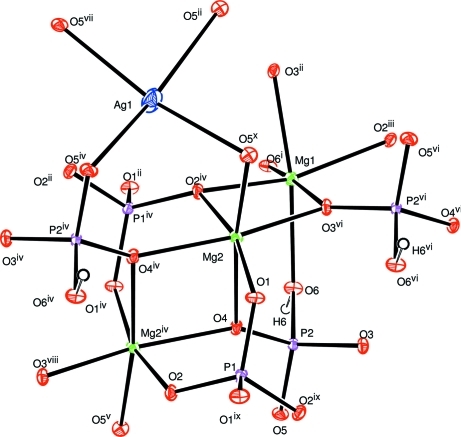
Partial plot of AgMg3(PO4)(HPO4)2 crystal structure. Displacement ellipsoids are drawn at the 50% probability level. Only the major component of the disordered silver atom is shown. Symmetry codes: (i) -x + 1/2, y - 1/2, -z + 1/2; (ii) x + 1/2, y - 1/2, z + 1; (iii) x + 1/2, -y + 1/2, z + 1/2; (iv) -x + 1/2, -y + 1/2, -z + 1; (v) -x + 1, y, -z + 3/2; (vi) -x + 1, -y, -z + 1; (vii) -x + 1, -y, -z + 2; (viii) x + 1/2, -y + 1/2, z - 1/2; (ix) -x + 1/2, -y + 1/2, -z; (x) -x + 1, y, -z + 1/2; (xi) -x, y, -z + 1/2; (xii) -x + 1/2, y + 1/2, -z + 1/2; (xiii) x - 1/2, y + 1/2, z - 1.

**Fig. 2.**
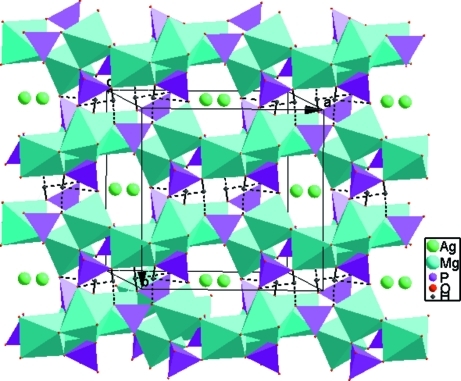
A three-dimensional polyhedral view of the crystal structure of the AgMg3(PO4)(HPO4)2, showing the channels running along the c direction,at 0,0,z and 1/2,0,z. Hydrogen bonds are indicated by dashed lines.

## supplementary crystallographic information

### Comment

Compounds belonging to the large structural family of alluaudite derivatives (Moore (1971); Hatert *et al.* (2000)) have been of continuing interest due to their structural properties, such as their open-framework architecture and their physical properties. Accordingly, the alluaudite structure exhibit an appropriate frameworks for a variety of applications, such as corrosion inhibition, passivation of metal surfaces, and catalysis (Hatert (2008); Korzenski *et al.* (1998); Kacimi *et al.* (2005)).

In addition, the accommodation of the monovalent cations in the one-dimensional channels of the alluaudite-like structures is strongly required for conductivity properties and have offered a great field of application as positive electrode in the lithium and sodium batteries (Trad *et al.* 2010)

By means of the powerful hydrothermal technique, our attempts to synthesize new monovalent divalent cations phosphate with alluaudite –like structure have successfully allowed to obtain a new silver magnesium phosphate phase. The present paper aims to report detailed hydrothermal synthesis and structural characterization of the title compound.

The structure is built up from MgO_6_ octahedra, PO_4_ and PO_3_(OH) tetrahedra, sharing corners and edges to form a three-dimensional framework as schown in Fig.1 and Fig.2. The three-dimensional network delimits two types of hexagonal channels which accommodate Ag+ cations and OH groups (see Fig.2). In the channels, each silver atoms is surrounded by four O atoms with Ag–O bond length varies between 2.3621 and 2.5150 Å. The same Ag^+^coordination sphere is observed in γ-AgZnPO_4_ (Assani *et al.* (2010)). Moreover the OH groups, pointing into one type of channel, are involved in strong hydrogen bonds. The silver trimagnesium phosphate bis-(hydrogenphosphate): AgMg_3_(PO_4_)(HPO_4_)_2_, is isostructural with the compounds A*M*_3_H_2_(XO_4_)_3_ (A = Na or Ag, *M* = Mn, Co or Ni, and *X* = P or As) of the alluaudite structure type (Guesmi & Driss (2002); Ben Smail & Jouini (2002); Stock & Bein (2003).

### Experimental

The crystals of the title compound has been hydrothermally synthesized starting from a mixture of magnesium oxide (0,0605 g), silver nitrate (0,1699 g), 85 wt % phosphoric acid (0,10 ml), and 12 ml of water. The hydrothermal synthesis was carried out in 23 ml Teflon-lined autoclave under autogeneous pressure at 468 K during 24 h. The product was filtered off, washed with deionized water and air dried. The reaction product consists yellow powder besides a colorless parallelepipedic crystals of the title compound.

### Refinement

The O-bound H atoms were initially located in a difference map and refined with O—H distance restraints of 0.86 (1), for the water molecule. In the last cycle they were refined in the riding model approximation with *U*_iso_(H) set to 1.5*U*_eq_(O).

In this model of the title compound, the atomic displacement parameters for Ag are higher than those of other atoms. This is due to the fact that Ag is in a channel. The same phenomenon is observed in the case of crystal structures of AgCo_3_(PO_4_)(HPO_4_)_2_; AgNi_3_(PO_4_)(HPO_4_)_2_ and AgMn_3_(AsO_4_)(HAsO_4_)_2_. However, Leroux *et al.* (1995) have proposed another model in the case of AgMn_3_(PO_4_)(HPO_4_)_2_ in which Ag is split into two very near sites with relatively weak atomic displacement parameters. The refinement is slightly better in this model.

### Crystal data

**Table:**

AgMg_3_(PO_4_)(HPO_4_)_2_	*F*(000) = 904
*M**_r_* = 467.73	*D*_x_ = 3.631 Mg m^−^^3^
Monoclinic, *C*2/*c*	Mo *K*α radiation, λ = 0.71073 Å
Hall symbol: -C 2yc	Cell parameters from 2330 reflections
*a* = 11.9126 (5) Å	θ = 2.5–38.0°
*b* = 12.1197 (6) Å	µ = 3.21 mm^−^^1^
*c* = 6.4780 (3) Å	*T* = 296 K
β = 113.812 (2)°	Prism, colourless
*V* = 855.66 (7) Å^3^	0.31 × 0.16 × 0.12 mm
*Z* = 4	

### Data collection

**Table:**

Bruker X8 APEX diffractometer	2330 independent reflections
Radiation source: fine-focus sealed tube	1998 reflections with *I* > 2σ(*I*)
graphite	*R*_int_ = 0.034
φ and ω scans	θ_max_ = 38.0°, θ_min_ = 2.5°
Absorption correction: multi-scan (*SADABS*; Bruker, 2005)	*h* = −20→20
*T*_min_ = 0.545, *T*_max_ = 0.680	*k* = −20→20
10680 measured reflections	*l* = −11→10

### Refinement

**Table:**

Refinement on *F*^2^	Secondary atom site location: difference Fourier map
Least-squares matrix: full	Hydrogen site location: inferred from neighbouring sites
*R*[*F*^2^ > 2σ(*F*^2^)] = 0.026	H-atom parameters constrained
*wR*(*F*^2^) = 0.075	*w* = 1/[σ^2^(*F*_o_^2^) + (0.0323*P*)^2^ + 1.8049*P*] where *P* = (*F*_o_^2^ + 2*F*_c_^2^)/3
*S* = 1.08	(Δ/σ)_max_ = 0.002
2330 reflections	Δρ_max_ = 0.63 e Å^−^^3^
91 parameters	Δρ_min_ = −1.28 e Å^−^^3^
0 restraints	Extinction correction: *SHELXL97* (Sheldrick, 2008), Fc^*^=kFc[1+0.001xFc^2^λ^3^/sin(2θ)]^-1/4^
Primary atom site location: structure-invariant direct methods	Extinction coefficient: 0.0013 (3)

### Special details

**Table:**

Geometry. All e.s.d.'s (except the e.s.d. in the dihedral angle between two l.s. planes) are estimated using the full covariance matrix. The cell e.s.d.'s are taken into account individually in the estimation of e.s.d.'s in distances, angles and torsion angles; correlations between e.s.d.'s in cell parameters are only used when they are defined by crystal symmetry. An approximate (isotropic) treatment of cell e.s.d.'s is used for estimating e.s.d.'s involving l.s. planes.
Refinement. Refinement of *F*^2^ against ALL reflections. The weighted *R*-factor *wR* and goodness of fit *S* are based on *F*^2^, conventional *R*-factors *R* are based on *F*, with *F* set to zero for negative *F*^2^. The threshold expression of *F*^2^ > σ(*F*^2^) is used only for calculating *R*-factors(gt) *etc*. and is not relevant to the choice of reflections for refinement. *R*-factors based on *F*^2^ are statistically about twice as large as those based on *F*, and *R*- factors based on ALL data will be even larger.

### Fractional atomic coordinates and isotropic or equivalent isotropic displacement parameters (Å^2^)

**Table:**

	*x*	*y*	*z*	*U*_iso_*/*U*_eq_	Occ. (<1)
Ag1A	0.5000	0.0261 (4)	0.7500	0.0192 (3)	0.89 (3)
Ag1B	0.5000	0.0047 (18)	0.7500	0.0192 (3)	0.11 (3)
Mg1	0.5000	0.27737 (7)	0.2500	0.00765 (14)	
Mg2	0.28999 (6)	0.16182 (5)	0.37691 (10)	0.00621 (10)	
P1	0.0000	0.18606 (5)	0.2500	0.00545 (10)	
P2	0.22298 (4)	0.38713 (3)	0.11567 (7)	0.00508 (8)	
O1	0.10721 (11)	0.10964 (10)	0.2643 (2)	0.00793 (19)	
O2	0.03617 (10)	0.25753 (10)	0.46302 (18)	0.00686 (19)	
O3	0.15657 (11)	0.32826 (10)	−0.11097 (19)	0.00676 (19)	
O4	0.21721 (11)	0.31920 (10)	0.30907 (18)	0.00623 (18)	
O5	0.16491 (11)	0.50095 (10)	0.1050 (2)	0.00777 (19)	
O6	0.36178 (11)	0.40491 (10)	0.1603 (2)	0.00812 (19)	
H6	0.3747	0.4749	0.1705	0.012*	

### Atomic displacement parameters (Å^2^)

**Table:**

	*U*^11^	*U*^22^	*U*^33^	*U*^12^	*U*^13^	*U*^23^
Ag1A	0.00987 (9)	0.0318 (8)	0.01280 (10)	0.000	0.00135 (7)	0.000
Ag1B	0.00987 (9)	0.0318 (8)	0.01280 (10)	0.000	0.00135 (7)	0.000
Mg1	0.0087 (3)	0.0072 (3)	0.0080 (3)	0.000	0.0044 (3)	0.000
Mg2	0.0076 (2)	0.0049 (2)	0.0068 (2)	0.00050 (17)	0.00358 (18)	0.00038 (17)
P1	0.0062 (2)	0.0052 (2)	0.0043 (2)	0.000	0.00141 (17)	0.000
P2	0.00704 (15)	0.00373 (16)	0.00436 (15)	0.00011 (11)	0.00219 (12)	0.00006 (11)
O1	0.0057 (4)	0.0061 (5)	0.0116 (5)	0.0009 (3)	0.0032 (4)	−0.0004 (4)
O2	0.0064 (4)	0.0087 (5)	0.0049 (4)	−0.0008 (3)	0.0017 (3)	−0.0018 (3)
O3	0.0086 (5)	0.0069 (5)	0.0045 (4)	−0.0009 (3)	0.0023 (3)	−0.0011 (3)
O4	0.0083 (4)	0.0058 (4)	0.0048 (4)	0.0001 (3)	0.0029 (3)	0.0013 (3)
O5	0.0091 (5)	0.0045 (4)	0.0096 (5)	0.0012 (3)	0.0036 (4)	−0.0004 (3)
O6	0.0067 (4)	0.0056 (5)	0.0123 (5)	−0.0007 (3)	0.0042 (4)	−0.0001 (4)

### Geometric parameters (Å, °)

**Table:**

Ag1A—O5^i^	2.3649 (14)	Mg2—O5^i^	2.0132 (13)
Ag1A—O5^ii^	2.3649 (14)	Mg2—O3^ix^	2.0672 (13)
Ag1A—O5^iii^	2.5177 (14)	Mg2—O4	2.0677 (13)
Ag1A—O5^iv^	2.5177 (14)	Mg2—O4^iv^	2.0831 (13)
Ag1A—Mg2	3.150 (3)	Mg2—O1	2.0954 (13)
Ag1A—Mg2^v^	3.150 (3)	Mg2—O2^iv^	2.1414 (13)
Ag1A—Ag1B^vi^	3.260 (3)	Mg2—Mg2^iv^	3.0408 (12)
Ag1A—Ag1B^vii^	3.260 (3)	Mg2—P2	3.1411 (7)
Ag1A—Ag1A^vi^	3.3001 (19)	Mg2—P2^ix^	3.1874 (7)
Ag1A—Ag1A^vii^	3.3001 (19)	P1—O2	1.5363 (12)
Ag1B—O5^i^	2.3457 (13)	P1—O2^xi^	1.5363 (12)
Ag1B—O5^ii^	2.3457 (13)	P1—O1	1.5497 (12)
Ag1B—O5^iii^	2.4972 (14)	P1—O1^xi^	1.5497 (12)
Ag1B—O5^iv^	2.4972 (14)	P2—O4	1.5237 (12)
Ag1B—Ag1B^vi^	3.2410 (15)	P2—O5	1.5322 (13)
Ag1B—Ag1B^vii^	3.2410 (15)	P2—O3	1.5337 (12)
Ag1B—Ag1A^vi^	3.260 (3)	P2—O6	1.5742 (13)
Ag1B—Ag1A^vii^	3.260 (3)	P2—Mg2^ix^	3.1874 (7)
Ag1B—Mg2	3.293 (12)	O2—Mg1^iv^	2.1136 (12)
Ag1B—Mg2^v^	3.293 (12)	O2—Mg2^iv^	2.1414 (13)
Ag1B—Mg1^vi^	3.42 (2)	O3—Mg2^ix^	2.0672 (13)
Mg1—O2^viii^	2.1136 (12)	O3—Mg1^ix^	2.1384 (13)
Mg1—O2^iv^	2.1136 (12)	O4—Mg2^iv^	2.0831 (13)
Mg1—O3^ix^	2.1383 (13)	O5—Mg2^xii^	2.0133 (13)
Mg1—O3^iii^	2.1383 (13)	O5—Ag1B^xiii^	2.3457 (13)
Mg1—O6	2.1600 (14)	O5—Ag1A^xiii^	2.3649 (14)
Mg1—O6^x^	2.1601 (14)	O5—Ag1B^iv^	2.4972 (14)
Mg1—Mg2^x^	3.2491 (7)	O5—Ag1A^iv^	2.5177 (14)
Mg1—Mg2	3.2492 (7)	O6—H6	0.8600
Mg1—Ag1B^vi^	3.42 (2)		
			
O5^i^—Ag1A—O5^ii^	165.2 (2)	O6^x^—Mg1—Mg2	145.25 (4)
O5^i^—Ag1A—O5^iii^	95.01 (5)	Mg2^x^—Mg1—Mg2	128.94 (3)
O5^ii^—Ag1A—O5^iii^	83.06 (5)	O2^viii^—Mg1—Ag1B^vi^	78.46 (4)
O5^i^—Ag1A—O5^iv^	83.06 (5)	O2^iv^—Mg1—Ag1B^vi^	78.46 (4)
O5^ii^—Ag1A—O5^iv^	95.01 (5)	O3^ix^—Mg1—Ag1B^vi^	53.22 (4)
O5^iii^—Ag1A—O5^iv^	165.0 (2)	O3^iii^—Mg1—Ag1B^vi^	53.22 (4)
O5^i^—Ag1A—Mg2	39.70 (5)	O6—Mg1—Ag1B^vi^	135.69 (4)
O5^ii^—Ag1A—Mg2	154.68 (19)	O6^x^—Mg1—Ag1B^vi^	135.70 (4)
O5^iii^—Ag1A—Mg2	106.22 (6)	Mg2^x^—Mg1—Ag1B^vi^	64.468 (17)
O5^iv^—Ag1A—Mg2	81.75 (5)	Mg2—Mg1—Ag1B^vi^	64.467 (17)
O5^i^—Ag1A—Mg2^v^	154.68 (19)	O5^i^—Mg2—O3^ix^	86.56 (5)
O5^ii^—Ag1A—Mg2^v^	39.70 (5)	O5^i^—Mg2—O4	170.04 (6)
O5^iii^—Ag1A—Mg2^v^	81.75 (5)	O3^ix^—Mg2—O4	90.74 (5)
O5^iv^—Ag1A—Mg2^v^	106.22 (6)	O5^i^—Mg2—O4^iv^	99.52 (5)
Mg2—Ag1A—Mg2^v^	117.04 (15)	O3^ix^—Mg2—O4^iv^	162.82 (6)
O5^i^—Ag1A—Ag1B^vi^	49.64 (5)	O4—Mg2—O4^iv^	85.79 (5)
O5^ii^—Ag1A—Ag1B^vi^	128.18 (15)	O5^i^—Mg2—O1	86.75 (5)
O5^iii^—Ag1A—Ag1B^vi^	45.70 (5)	O3^ix^—Mg2—O1	110.73 (5)
O5^iv^—Ag1A—Ag1B^vi^	131.97 (16)	O4—Mg2—O1	85.23 (5)
Mg2—Ag1A—Ag1B^vi^	67.4 (3)	O4^iv^—Mg2—O1	85.78 (5)
Mg2^v^—Ag1A—Ag1B^vi^	120.2 (3)	O5^i^—Mg2—O2^iv^	103.34 (5)
O5^i^—Ag1A—Ag1B^vii^	128.18 (15)	O3^ix^—Mg2—O2^iv^	79.28 (5)
O5^ii^—Ag1A—Ag1B^vii^	49.64 (5)	O4—Mg2—O2^iv^	85.55 (5)
O5^iii^—Ag1A—Ag1B^vii^	131.97 (16)	O4^iv^—Mg2—O2^iv^	83.67 (5)
O5^iv^—Ag1A—Ag1B^vii^	45.70 (5)	O1—Mg2—O2^iv^	166.46 (6)
Mg2—Ag1A—Ag1B^vii^	120.2 (3)	O5^i^—Mg2—Mg2^iv^	141.54 (5)
Mg2^v^—Ag1A—Ag1B^vii^	67.4 (3)	O3^ix^—Mg2—Mg2^iv^	131.55 (5)
Ag1B^vi^—Ag1A—Ag1B^vii^	166.9 (9)	O4—Mg2—Mg2^iv^	43.09 (3)
O5^i^—Ag1A—Ag1A^vi^	49.46 (4)	O4^iv^—Mg2—Mg2^iv^	42.70 (3)
O5^ii^—Ag1A—Ag1A^vi^	126.91 (10)	O1—Mg2—Mg2^iv^	83.86 (4)
O5^iii^—Ag1A—Ag1A^vi^	45.55 (3)	O2^iv^—Mg2—Mg2^iv^	82.63 (4)
O5^iv^—Ag1A—Ag1A^vi^	130.57 (10)	O5^i^—Mg2—P2	151.29 (4)
Mg2—Ag1A—Ag1A^vi^	70.23 (7)	O3^ix^—Mg2—P2	66.25 (4)
Mg2^v^—Ag1A—Ag1A^vi^	122.57 (9)	O4—Mg2—P2	24.50 (3)
Ag1B^vi^—Ag1A—Ag1A^vi^	4.5 (3)	O4^iv^—Mg2—P2	109.17 (4)
Ag1B^vii^—Ag1A—Ag1A^vi^	162.4 (6)	O1—Mg2—P2	94.16 (4)
O5^i^—Ag1A—Ag1A^vii^	126.91 (10)	O2^iv^—Mg2—P2	81.36 (4)
O5^ii^—Ag1A—Ag1A^vii^	49.46 (4)	Mg2^iv^—Mg2—P2	66.81 (2)
O5^iii^—Ag1A—Ag1A^vii^	130.57 (10)	O5^i^—Mg2—Ag1A	48.62 (8)
O5^iv^—Ag1A—Ag1A^vii^	45.55 (3)	O3^ix^—Mg2—Ag1A	104.68 (4)
Mg2—Ag1A—Ag1A^vii^	122.57 (9)	O4—Mg2—Ag1A	141.24 (8)
Mg2^v^—Ag1A—Ag1A^vii^	70.23 (7)	O4^iv^—Mg2—Ag1A	68.99 (5)
Ag1B^vi^—Ag1A—Ag1A^vii^	162.4 (6)	O1—Mg2—Ag1A	120.08 (7)
Ag1B^vii^—Ag1A—Ag1A^vii^	4.5 (3)	O2^iv^—Mg2—Ag1A	63.49 (8)
Ag1A^vi^—Ag1A—Ag1A^vii^	157.9 (3)	Mg2^iv^—Mg2—Ag1A	106.54 (6)
O5^i^—Ag1B—O5^ii^	177.8 (10)	P2—Mg2—Ag1A	144.85 (7)
O5^i^—Ag1B—O5^iii^	96.05 (5)	O5^i^—Mg2—P2^ix^	77.41 (4)
O5^ii^—Ag1B—O5^iii^	83.89 (5)	O3^ix^—Mg2—P2^ix^	23.55 (3)
O5^i^—Ag1B—O5^iv^	83.89 (5)	O4—Mg2—P2^ix^	96.44 (4)
O5^ii^—Ag1B—O5^iv^	96.05 (5)	O4^iv^—Mg2—P2^ix^	173.57 (4)
O5^iii^—Ag1B—O5^iv^	176.9 (10)	O1—Mg2—P2^ix^	88.39 (4)
O5^i^—Ag1B—Ag1B^vi^	50.02 (3)	O2^iv^—Mg2—P2^ix^	102.49 (4)
O5^ii^—Ag1B—Ag1B^vi^	129.88 (8)	Mg2^iv^—Mg2—P2^ix^	139.20 (3)
O5^iii^—Ag1B—Ag1B^vi^	46.03 (3)	P2—Mg2—P2^ix^	73.940 (17)
O5^iv^—Ag1B—Ag1B^vi^	133.82 (10)	Ag1A—Mg2—P2^ix^	111.92 (4)
O5^i^—Ag1B—Ag1B^vii^	129.88 (8)	O5^i^—Mg2—Mg1	102.56 (4)
O5^ii^—Ag1B—Ag1B^vii^	50.02 (3)	O3^ix^—Mg2—Mg1	40.22 (3)
O5^iii^—Ag1B—Ag1B^vii^	133.82 (10)	O4—Mg2—Mg1	81.27 (4)
O5^iv^—Ag1B—Ag1B^vii^	46.03 (3)	O4^iv^—Mg2—Mg1	122.61 (4)
Ag1B^vi^—Ag1B—Ag1B^vii^	176.0 (15)	O1—Mg2—Mg1	147.15 (4)
O5^i^—Ag1B—Ag1A^vi^	50.19 (5)	O2^iv^—Mg2—Mg1	39.90 (3)
O5^ii^—Ag1B—Ag1A^vi^	129.49 (14)	Mg2^iv^—Mg2—Mg1	105.43 (3)
O5^iii^—Ag1B—Ag1A^vi^	46.19 (4)	P2—Mg2—Mg1	62.814 (18)
O5^iv^—Ag1B—Ag1A^vi^	133.32 (16)	Ag1A—Mg2—Mg1	88.00 (4)
Ag1B^vi^—Ag1B—Ag1A^vi^	4.6 (3)	P2^ix^—Mg2—Mg1	63.765 (15)
Ag1B^vii^—Ag1B—Ag1A^vi^	171.4 (12)	O5^i^—Mg2—Ag1B	44.9 (3)
O5^i^—Ag1B—Ag1A^vii^	129.49 (14)	O3^ix^—Mg2—Ag1B	104.30 (5)
O5^ii^—Ag1B—Ag1A^vii^	50.19 (5)	O4—Mg2—Ag1B	144.9 (3)
O5^iii^—Ag1B—Ag1A^vii^	133.32 (16)	O4^iv^—Mg2—Ag1B	70.47 (13)
O5^iv^—Ag1B—Ag1A^vii^	46.19 (4)	O1—Mg2—Ag1B	117.1 (2)
Ag1B^vi^—Ag1B—Ag1A^vii^	171.4 (12)	O2^iv^—Mg2—Ag1B	67.0 (3)
Ag1B^vii^—Ag1B—Ag1A^vii^	4.6 (3)	Mg2^iv^—Mg2—Ag1B	109.1 (2)
Ag1A^vi^—Ag1B—Ag1A^vii^	166.9 (9)	P2—Mg2—Ag1B	148.3 (3)
O5^i^—Ag1B—Mg2	37.3 (2)	Ag1A—Mg2—Ag1B	3.9 (2)
O5^ii^—Ag1B—Mg2	144.9 (8)	P2^ix^—Mg2—Ag1B	110.03 (15)
O5^iii^—Ag1B—Mg2	102.7 (3)	Mg1—Mg2—Ag1B	90.03 (16)
O5^iv^—Ag1B—Mg2	79.2 (2)	O2—P1—O2^xi^	111.36 (10)
Ag1B^vi^—Ag1B—Mg2	66.0 (4)	O2—P1—O1	110.96 (6)
Ag1B^vii^—Ag1B—Mg2	116.6 (6)	O2^xi^—P1—O1	108.44 (6)
Ag1A^vi^—Ag1B—Mg2	69.01 (18)	O2—P1—O1^xi^	108.44 (6)
Ag1A^vii^—Ag1B—Mg2	119.4 (3)	O2^xi^—P1—O1^xi^	110.95 (6)
O5^i^—Ag1B—Mg2^v^	144.9 (8)	O1—P1—O1^xi^	106.60 (10)
O5^ii^—Ag1B—Mg2^v^	37.3 (2)	O4—P2—O5	110.74 (7)
O5^iii^—Ag1B—Mg2^v^	79.2 (2)	O4—P2—O3	111.02 (7)
O5^iv^—Ag1B—Mg2^v^	102.7 (3)	O5—P2—O3	109.06 (7)
Ag1B^vi^—Ag1B—Mg2^v^	116.6 (6)	O4—P2—O6	108.40 (7)
Ag1B^vii^—Ag1B—Mg2^v^	66.0 (4)	O5—P2—O6	107.86 (7)
Ag1A^vi^—Ag1B—Mg2^v^	119.4 (3)	O3—P2—O6	109.70 (7)
Ag1A^vii^—Ag1B—Mg2^v^	69.01 (18)	O4—P2—Mg2	34.24 (5)
Mg2—Ag1B—Mg2^v^	109.3 (6)	O5—P2—Mg2	144.97 (5)
O5^i^—Ag1B—Mg1^vi^	88.9 (5)	O3—P2—Mg2	91.84 (5)
O5^ii^—Ag1B—Mg1^vi^	88.9 (5)	O6—P2—Mg2	90.03 (5)
O5^iii^—Ag1B—Mg1^vi^	88.4 (5)	O4—P2—Mg2^ix^	136.27 (5)
O5^iv^—Ag1B—Mg1^vi^	88.4 (5)	O5—P2—Mg2^ix^	106.49 (5)
Ag1B^vi^—Ag1B—Mg1^vi^	88.0 (8)	O3—P2—Mg2^ix^	32.58 (5)
Ag1B^vii^—Ag1B—Mg1^vi^	88.0 (8)	O6—P2—Mg2^ix^	80.34 (5)
Ag1A^vi^—Ag1B—Mg1^vi^	83.4 (5)	Mg2—P2—Mg2^ix^	106.060 (17)
Ag1A^vii^—Ag1B—Mg1^vi^	83.4 (5)	P1—O1—Mg2	123.77 (7)
Mg2—Ag1B—Mg1^vi^	125.3 (3)	P1—O2—Mg1^iv^	126.47 (7)
Mg2^v^—Ag1B—Mg1^vi^	125.3 (3)	P1—O2—Mg2^iv^	123.92 (7)
O2^viii^—Mg1—O2^iv^	156.91 (8)	Mg1^iv^—O2—Mg2^iv^	99.56 (5)
O2^viii^—Mg1—O3^ix^	87.86 (5)	P2—O3—Mg2^ix^	123.87 (7)
O2^iv^—Mg1—O3^ix^	78.33 (5)	P2—O3—Mg1^ix^	134.97 (7)
O2^viii^—Mg1—O3^iii^	78.33 (5)	Mg2^ix^—O3—Mg1^ix^	101.16 (5)
O2^iv^—Mg1—O3^iii^	87.86 (5)	P2—O4—Mg2	121.26 (7)
O3^ix^—Mg1—O3^iii^	106.45 (7)	P2—O4—Mg2^iv^	140.95 (7)
O2^viii^—Mg1—O6	108.09 (5)	Mg2—O4—Mg2^iv^	94.21 (5)
O2^iv^—Mg1—O6	88.61 (5)	P2—O5—Mg2^xii^	139.84 (8)
O3^ix^—Mg1—O6	82.80 (4)	P2—O5—Ag1B^xiii^	104.2 (5)
O3^iii^—Mg1—O6	169.20 (5)	Mg2^xii^—O5—Ag1B^xiii^	97.9 (5)
O2^viii^—Mg1—O6^x^	88.61 (5)	P2—O5—Ag1A^xiii^	110.03 (12)
O2^iv^—Mg1—O6^x^	108.09 (5)	Mg2^xii^—O5—Ag1A^xiii^	91.67 (12)
O3^ix^—Mg1—O6^x^	169.20 (5)	Ag1B^xiii^—O5—Ag1A^xiii^	6.3 (4)
O3^iii^—Mg1—O6^x^	82.80 (4)	P2—O5—Ag1B^iv^	111.6 (5)
O6—Mg1—O6^x^	88.61 (7)	Mg2^xii^—O5—Ag1B^iv^	103.7 (5)
O2^viii^—Mg1—Mg2^x^	40.53 (3)	Ag1B^xiii^—O5—Ag1B^iv^	83.95 (5)
O2^iv^—Mg1—Mg2^x^	125.98 (4)	Ag1A^xiii^—O5—Ag1B^iv^	84.17 (8)
O3^ix^—Mg1—Mg2^x^	105.38 (4)	P2—O5—Ag1A^iv^	105.79 (12)
O3^iii^—Mg1—Mg2^x^	38.62 (3)	Mg2^xii^—O5—Ag1A^iv^	109.53 (12)
O6—Mg1—Mg2^x^	145.25 (4)	Ag1B^xiii^—O5—Ag1A^iv^	84.12 (8)
O6^x^—Mg1—Mg2^x^	78.19 (3)	Ag1A^xiii^—O5—Ag1A^iv^	84.99 (5)
O2^viii^—Mg1—Mg2	125.98 (4)	Ag1B^iv^—O5—Ag1A^iv^	5.9 (4)
O2^iv^—Mg1—Mg2	40.53 (3)	P2—O6—Mg1	125.56 (7)
O3^ix^—Mg1—Mg2	38.62 (3)	P2—O6—H6	106.9
O3^iii^—Mg1—Mg2	105.38 (4)	Mg1—O6—H6	126.3
O6—Mg1—Mg2	78.19 (3)		

Symmetry codes: (i) −*x*+1/2, *y*−1/2, −*z*+1/2; (ii) *x*+1/2, *y*−1/2, *z*+1; (iii) *x*+1/2, −*y*+1/2, *z*+1/2; (iv) −*x*+1/2, −*y*+1/2, −*z*+1; (v) −*x*+1, *y*, −*z*+3/2; (vi) −*x*+1, −*y*, −*z*+1; (vii) −*x*+1, −*y*, −*z*+2; (viii) *x*+1/2, −*y*+1/2, *z*−1/2; (ix) −*x*+1/2, −*y*+1/2, −*z*; (x) −*x*+1, *y*, −*z*+1/2; (xi) −*x*, *y*, −*z*+1/2; (xii) −*x*+1/2, *y*+1/2, −*z*+1/2; (xiii) *x*−1/2, *y*+1/2, *z*−1.

### Hydrogen-bond geometry (Å, °)

**Table:**

*D*—H···*A*	*D*—H	H···*A*	*D*···*A*	*D*—H···*A*
O6—H6···O1^xiv^	0.86	1.68	2.5266 (17)	168

Symmetry codes: (xiv) −*x*+1/2, *y*+1/2, −*z*+1/2.
